# Educational Differences in Sexual Health among Adolescents in Europe: A Meta-Analysis

**DOI:** 10.1007/s10964-026-02364-3

**Published:** 2026-06-08

**Authors:** Pascalle Heijligenberg, Margreet E. de Looze, Marlies Maes, Kai J. Jonas, Karlijn Massar

**Affiliations:** 1 Department of Work and Social Psychology, Faculty of Psychology and Neuroscience, Maastricht University, Maastricht, Netherlands; 2 Department of Interdisciplinary Social Science, Faculty of Social and Behavioural Sciences, Utrecht University, Utrecht, Netherlands

**Keywords:** Adolescents, Education, Sexual health, Meta-analysis

## Abstract

In European countries with stratified educational trajectories, in which adolescents are separated into vocational and academic tracks, studies have shown that there are educational differences in adolescent sexual health. However, due to heterogeneity of the existing literature in terms of national educational systems and dimensions of sexual health being studied, uncertainty remains about the strength of the association between educational track and adolescent sexual health. The current meta-analysis synthesizes the available evidence on educational differences in sexual health among adolescents in Europe. Further, it tests the extent to which findings vary according to the (sub)domain of sexual health being studied, and study and sample characteristics such as study quality, gender and ethnic background of participants. This meta-analysis included 47 studies, with 159 effect sizes, and 177,169 adolescents (*M*_*age*_ = 16.59, *SD* = 2.17). Results revealed a significant association between educational track and adolescent sexual health (*g* = 0.279, 95% CI[0.204, 0.354], *p* < 0.001). Associations were significantly stronger for certain (sub)domains of sexual health, with adolescents attending vocational tracks for example having less sexual knowledge, more often having had sex (at an early age) and more often experiencing teenage pregnancies, compared to adolescents attending academic tracks. Additionally, the association between educational track and adolescent sexual health was significantly stronger in studies in which more than 75% of participants did not have a migration background. However, there was high heterogeneity (*Q*(158) = 1960.297, *p* < 0.001; *I*^*2*^ = 80.23%) across studies, which remained significant after adding the moderators, indicating substantial complexity in this relationship that warrants cautious interpretation. This meta-analysis confirmed that there are educational track differences in sexual health among adolescents in Europe, and clarified how this relationship varies according to sexual health (sub)domains and study and sample characteristics.

**Supplementary Information:**

The online version contains supplementary material available at 10.1007/s10964-026-02364-3.

## Introduction

Adolescence is a critical period for the development of sexual knowledge, attitudes, and behaviors (Tolman & McClelland, 2011), and disparities emerging during this stage may contribute to longer-term inequalities in sexual health (Slurink et al., 2021; Vasilenko, 2022; Warzecha et al., 2019). Educational systems may shape such disparities, especially through educational tracking, in which students are separated into academic or vocational tracks (OECD, 2024). Several studies across European countries with tracked educational systems have shown that students in vocational tracks tend to report more sexual risk behavior and less sexual health knowledge than students in academic tracks (e.g., De Graaf et al., 2015; Di Gennaro et al., 2024; Häggström-Nordin et al., 2011; Patseadou et al., 2010; van Rossem et al., 2010). However, given the heterogeneity of the existing literature - in terms of national educational systems and dimensions of sexual health being studied - it is difficult to draw general conclusions about the association between educational track and adolescent sexual health. Clarifying whether these disparities are consistent across different contexts, and which aspects of sexual health are most strongly associated with educational track, is important for understanding the extent and nature of this relationship. A robust association would suggest systematic differences that warrant attention and potential intervention. Such insights could inform the development of more effective sexual health education and prevention strategies tailored to adolescents in different educational pathways, potentially reducing long-term social inequalities in sexual health (Vasilenko, 2022). This meta-analysis addresses gaps in the understanding of the relationship between educational track and adolescent sexual health, synthesizing studies from diverse national contexts and considering multiple dimensions of sexual health.

### Conceptualizing Educational Tracking

Educational tracking refers to the process by which students are selected and placed into different educational tracks based on perceived ability, guiding subsequent transitions to further levels of education and work (Gogescu, 2024). Most studies assessing the association between educational track and adolescent sexual health have been conducted within a single national context and reflect the specific characteristics of that country’s educational system and sociocultural environment. Educational tracking systems, however, vary considerably across countries, which may affect the comparability of studies in this meta-analysis. Specifically, the age at which students are tracked and the number and nature of tracks differ across national contexts. In Europe, students typically enter secondary school between ages 10 and 12 and complete it by ages 16 to 19, although there is variation in the timing and length of secondary education across countries. In some countries, such as Germany, Austria, Switzerland, the Netherlands and Belgium, students are assigned to different educational tracks early, typically at the beginning of secondary school. In other countries, such as Sweden, Norway, Finland, Poland and the United Kingdom, students follow a comprehensive secondary education for a few years before they are tracked into distinct educational pathways. Furthermore, the number and nature of educational tracks also vary: some countries, such as Finland and Spain, provide only two main tracks—vocational and academic—while others, like France and Hungary, divide students into multiple pathways, including specialized technical, vocational, and academic tracks (OECD, 2024). These different educational tracks can still be broadly categorized as either vocational or academic, as they either prepare for further vocational education and training (VET), or lead to a university-entrance certificate with which students can proceed to higher education (OECD, 2024; UNESCO Institute for Statistics, 2012). In order to maintain conceptual clarity and ensure cross-national comparability, the educational tracks in each study were categorized as vocational or academic based on available descriptions of the national educational systems and the International Standard Classification of Education (ISCED; UNESCO Institute for Statistics, 2012). An overview of the classification approach is provided in the supplementary materials.

### Conceptualizing Sexual Health

Sexual health is defined and operationalized in various ways in the literature (Hogben et al., 2016). For the purposes of this meta-analysis, the working definition of the World Health Organization is used, as it provides a comprehensive and widely adopted framework of sexual health (Douglas & Fenton, 2013; Vasconcelos et al., 2024). According to this definition, sexual health is a multidimensional concept; it is “a state of physical, emotional, mental and social well-being related to sexuality; it is not merely the absence of disease, dysfunction or infirmity. Sexual health requires a positive and respectful approach to sexuality and sexual relationships, as well as the possibility of having pleasurable and safe sexual experiences, free of coercion, discrimination and violence” (World Health Organization, 2006, p. 5). Following this definition, a wide range of domains of sexual health is included in this study, including sexual behavior, attitudes and knowledge. Much of the literature on the association between educational track and adolescent sexual health has focused primarily on risk-related outcomes, such as early sexual initiation, unintended pregnancy, or sexually transmitted infections (STIs) (e.g., Di Gennaro et al., 2024; Häggström-Nordin et al., 2011; Patseadou et al., 2010; van Rossem et al., 2010), whereas fewer studies have examined broader or more positive dimensions of sexual health, including sexual attitudes, satisfaction, or relational and emotional aspects of sexuality (de Graaf et al., 2015; Seiler-Ramadas et al., 2020). Because individual studies typically assess only a limited set of outcomes, it remains unclear whether educational differences in sexual health are consistent across domains or whether they are specific to certain aspects of sexual health. A detailed description is provided of how sexual health was operationalized in each study, including the categorization in the domains of sexual behavior, attitudes or knowledge for this meta-analysis (see Table 2). This approach accounts for variation in the measurement of sexual health while allowing for meaningful comparisons across studies.

### Theoretical Perspectives on the Relationship Between Educational Track and Sexual Health

Several theoretical perspectives shed light on why there may be educational differences in adolescents’ sexual health. One perspective concerns the timing of life transitions and the accompanying shifts in social relationships. Adolescents in vocational tracks start several activities that are typically perceived as milestones in the transition to adulthood (e.g., entering the labor market, starting a family) at younger ages than their academic peers (Bertogg & Imdorf, 2023; Hampf & Woessmann, 2017). De Looze et al. (2013) reported that both expectations regarding the timing of these life transitions and the perception of sexual activity as a typical adult behavior partly explained the earlier sexual initiation among students in vocational tracks. Adolescents in vocational tracks also spent more time with their peers and shared less information about their whereabouts with their parents, compared to those in academic tracks, and this too contributed to explaining their earlier sexual initiation (De Looze et al., 2012). Additionally, adolescents in vocational tracks may orient themselves more strongly toward the peer group and turn to (risky) sexual behaviors to earn recognition and status among their peers, in lieu of educational achievements to acquire status (De Looze et al., 2013; Vanden Abeele et al., 2014). Vanden Abeele et al. (2014), for example, found that self-perceived popularity, need for popularity and peer pressure contributed to explaining the higher prevalence of online sexual behaviors (specifically sexting and mobile porn use) among adolescents in vocational tracks.

Another potential explanation for educational differences in adolescent sexual health is differences in the quality and quantity of sex education – both at school and at home. Generally, mainstream sex education programs are less tailored to the needs of adolescents in vocational tracks (Van Rossem et al., 2010), and hence less effective in achieving the intended results (Van Keulen et al., 2015; as cited in De Graaf et al., 2015). Additionally, students in vocational tracks tend to have more conservative views on sexuality, and these have been suggested to play a role in teachers’ reluctance to discuss certain topics with them (De Graaf et al., 2015). Further, Schouten and colleagues (2007) reported that adolescents in vocational tracks communicated less often with their parents about sexuality than adolescents in academic tracks.

### Moderation of Educational Differences in Sexual Health

#### Sexual Health Domains and Subdomains

Educational differences might vary across different domains and subdomains of sexual health. Based on the aforementioned definition of sexual health suggested by the World Health Organization (2006) and a meta-analysis on sexual health interventions (Becasen et al., 2015), the current study distinguishes the following domains of sexual health: sexual behavior, attitudes, and knowledge. The domain of sexual behavior encompasses sexual activity and both risky and protective sexual behaviors, including online sexual risk behaviors, contraceptive and condom use, and number of sexual partners, as well as adverse health outcomes (e.g., STIs, unintended pregnancy). The domain of sexual attitudes includes beliefs, opinions and feelings regarding sexuality or different sexual behaviors. The domain of sexual knowledge encompasses knowledge about sexual health – in other words, STIs and HIV/AIDS; cause, transmission, and prevention of infections; or pregnancy prevention (Becasen et al., 2015). For the purposes of this study, the term subdomains is used to describe more specific outcomes within the overarching domains. Thus, for example, condom use is a subdomain of sexual behavior, evaluation of sexual experience a subdomain of sexual attitudes, and knowledge about STIs a subdomain of sexual knowledge. An overview of the subdomains included in this study can be found in Table 2.

While research on risk-related sexual behavior subdomains has shown that adolescents attending vocational tracks tend to report more sexual risk behavior, such as an earlier onset of sexual activity and less contraceptive use, compared to those in academic tracks (e.g., Häggström-Nordin et al., 2011; Patseadou et al., 2010), sexual behavior subdomains that move beyond a purely risk-related focus remain largely understudied. In addition, there is limited research on sexual attitudes and knowledge. Therefore, these domains and subdomains of sexual health are examined in an exploratory manner.

#### Sample and Study Characteristics

Several sample and study characteristics might affect educational differences in adolescent sexual health. The following nine characteristics were examined: gender, stage of adolescence, SES and ethnic background of participants, the geographical scope, timing of educational tracking, publication year and study quality.

Regarding gender, several studies have shown that the strength of the association between educational track and sexual health differs between girls and boys. For instance, in a Dutch study, girls in a vocational track less often perceived their sexual debut as pleasant compared to girls in an academic track, while no significant educational difference was found for boys (De Graaf et al., 2015). Additionally, a Polish study found no significant educational differences for boys regarding condom use, but did find that girls in a vocational track less often used condoms compared to girls in an academic track (Czerwinski et al., 2018). Thus, sexual health disparities may vary for girls and boys.

Concerning other sample and study characteristics, no empirical studies have examined their potential moderating effect on the relationship between educational track and adolescent sexual health. Therefore, these potential moderators are examined in an exploratory fashion. Regarding stage of adolescence, the developmental period of adolescence encompasses a wide age range (10–19 years; World Health Organization, n.d.), which can be broadly divided into early (10–13 years), middle (14–16 years) and late (17–19 years) adolescence (Kar et al., 2015). As sexual activity becomes more common with increasing age (Gambadauro et al., 2018), sexual health disparities (including attitudes and knowledge) may become more pronounced in later stages of adolescence. Regarding SES; while educational attainment is often used as a proxy for SES, the SES of adolescents encompasses additional dimensions, such as parental education, income and occupation (Gautam et al., 2023), which may moderate the association between educational track and adolescent sexual health. Ethnic background may also have a moderating effect, as youth with a migration background often face distinct cultural norms and barriers to sexual health resources (Wayal et al., 2017), producing differences even within the same educational track. Regarding geographical scope, studies that sampled participants from just a single site or city might yield less representative results (leading to random error) than studies that sampled from multiple sites or cities in one area, or from multiple areas. Moreover, the timing of educational tracking may be of relevance; the age at which students are placed into educational tracks varies across countries (e.g., around 12 in the Netherlands, and around 16 in Sweden). When students are separated in educational tracks at an earlier age, the association with sexual health might be stronger, as a study comparing 22 European countries found that in countries in which students are tracked earlier, the gap in self-reported overall health is strengthened (Delaruelle et al., 2020). Regarding publication year, it may be expected that more recent studies report a weaker association between educational track and adolescent sexual health, as efforts have been made over time to reduce educational disparities in sexual health (Borneskog et al., 2021). Lastly, study quality will be examined as a potential moderator of the relationship between educational track and adolescent sexual health because methodological rigor influences the reliability and validity of reported effect sizes.

## Current Study

A better understanding of the relationship between educational track and adolescent sexual health requires addressing the gaps found in prior research. Previous studies have largely been confined to single national contexts and have examined only a limited set of sexual health outcomes and moderating factors. As a result, the overall strength of these associations remains unclear, underscoring the need for a meta-analytic synthesis. This study conducts a meta-analysis on the relationship between educational track and adolescent sexual health based on original studies from European countries. This study aims to synthesize empirical studies on this relationship and calculate the overall effect size of this association (Research Question 1). Furthermore, it also seeks to explore potential moderating factors that may explain the divergent findings regarding educational track and adolescent sexual health (Research Question 2). Based on theoretical perspectives and existing research findings, this study hypothesizes that the meta-analysis confirms a positive association between educational track and adolescent sexual health (Hypothesis 1). Additionally, it is hypothesized that the (sub)domain of sexual health, the gender, stage of adolescence, SES and ethnic background of participants, the geographical scope, timing of educational tracking, publication year and study quality affect the strength of the relationship between educational track and adolescent sexual health (Hypothesis 2).

## Methods

This meta-analysis was conducted and reported in accordance with the Preferred Reporting Items for Systematic Reviews and Meta-Analyses (PRISMA) guidelines (Page et al., 2021).

### Literature Search

The literature search was carried out in four databases (PubMed, PsycINFO, Web of Science, and Scopus) on May 12, 2023, and an additional search was carried out on January 27, 2025. The search terms were selected by identifying key terms related to educational track, sexual health outcomes, adolescents and European countries. The exact search terms used can be found in the supplementary materials. The search strategy was formulated in collaboration with a scientific information specialist of the Maastricht University Library.

### Inclusion and Exclusion Criteria

To be eligible for inclusion in this meta-analysis, a source needed to include an empirical study that examined the relationship between educational track and sexual health among adolescents in Europe. There were no restrictions on the types of study design eligible for inclusion. In case of longitudinal studies, statistical information from the first assessment was chosen. Exclusion criteria based on report characteristics were publication year (only eligible when published between 1 January 2003–27 January 2025), and language (only English or Dutch articles included).

Regarding educational tracking, studies were eligible if they differentiated between different educational tracks in secondary schools. Subsequently, only studies conducted in European countries (all European countries excluding the transcontinental countries Russia, Turkey, Azerbaijan, Georgia and Kazakhstan) with educational systems with different educational tracks in secondary school were eligible for inclusion. Further, studies were eligible when studying an adolescent population, or when the mean age of the sample was between 10 and 19 years (as per the World Health Organization; World Health Organization, n.d.). Since the title and abstract of articles often did not indicate whether educational differences were examined or if adolescents were studied, the full-texts of articles meeting all other inclusion criteria were screened.

For sexual health, the previously mentioned definition and the key elements suggested by the World Health Organization (2006, 2010) were used to determine eligibility of studies. Consequently, when studies examined one or multiple of the following sexual health outcomes, they were eligible for inclusion: sexual experiences, (online) sexual behaviors, (risk of) STIs (including condom use), (risk of) pregnancy (including contraceptive use) and abortion, sexual attitudes, sexual knowledge, sexual satisfaction and pleasure, and sexual coercion, discrimination and violence.

### Screening Process

Two reviewers performed the screening of studies: PH and SSS for the first search, and PH and LB for the second search. For each search, a random sample of 25% of titles and abstracts and, subsequently, full texts, was screened independently and blindly by both reviewers. Inter-rater reliability was consistently high between the reviewers across both stages (ranging from Cohen’s Kappa *κ* = 0.93 to *κ* = 0.95). Disagreements were resolved by discussing until agreement was reached. After obtaining this good interrater reliability, the remaining studies were screened by PH.

**Fig. 1:**
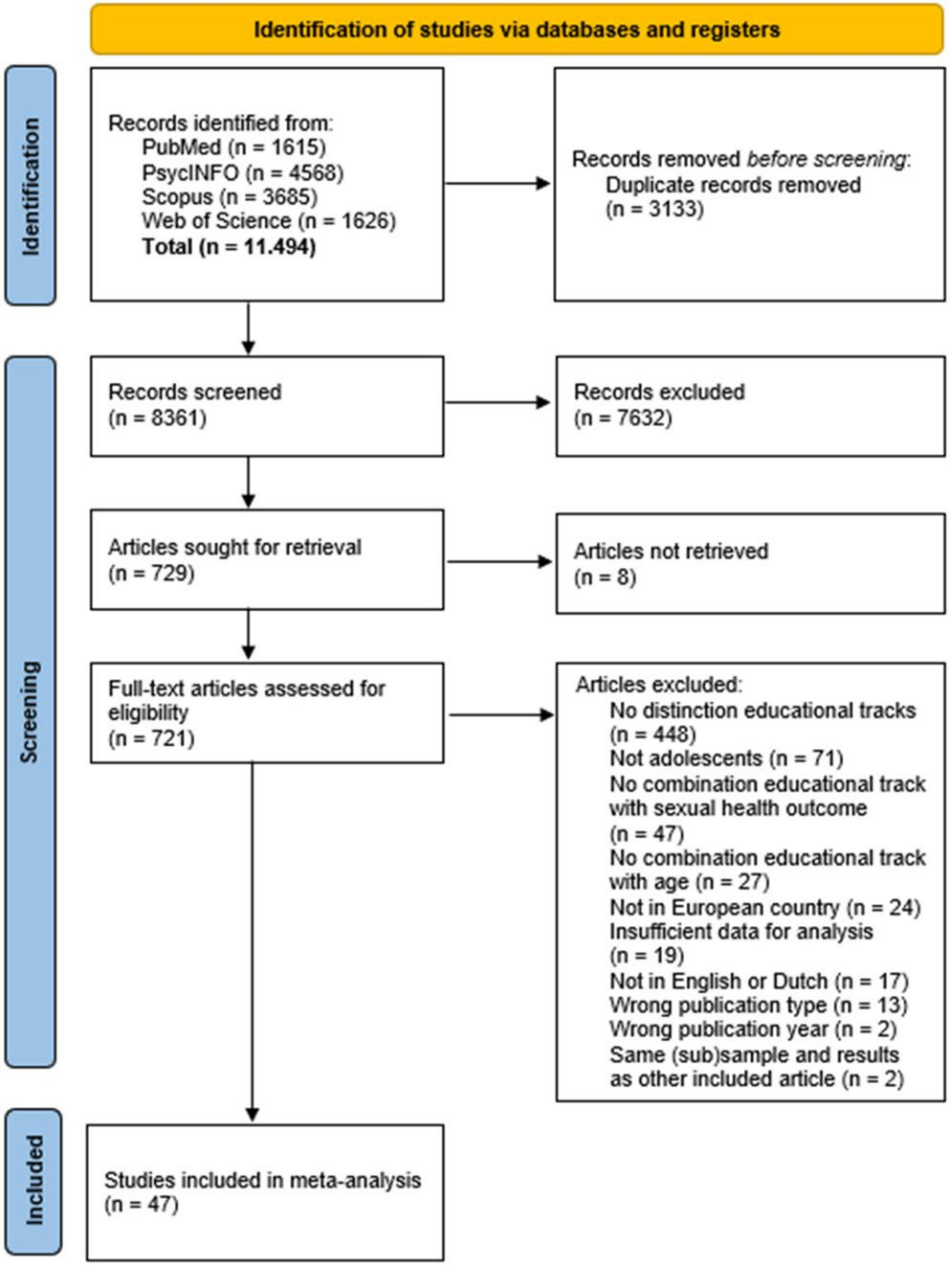
PRISMA Flow Diagram Showing Process of Study Selection

The data search initially yielded 8,361 non-duplicate hits (of which 7,243 from the first and 1,118 from the second search). The titles and abstracts of each individual study were reviewed using the Rayyan application (Ouzzani et al., 2016). 721 articles were included in the initial selection (of which 637 from the first and 84 from the second search), and a full-text review was conducted of those studies. In the end, 51 articles (of which 47 from the first and 4 from the second search; approximately 0.6% of the total screened titles and abstracts) were included. Five of these articles reported the same (sub)sample as five other articles but did report different measures. Thus, for the meta-analysis, these articles were merged to prevent overrepresentation of certain samples. Additionally, one article reported on two different studies with independent samples. Therefore, the meta-analysis is based on 51 articles, reporting 47 studies with independent samples, with a total of 159 effect sizes. The literature screening procedure is presented in Fig. 1.

### Data Extraction

The included studies were coded using a structured and pre-defined spreadsheet. Two reviewers (PH and MH) independently extracted and documented data from 30% of the included studies from the first search. As this led to no disagreements, PH extracted and documented data from the remaining studies. The present dataset includes 159 effect sizes (*k*) from 47 studies (*n*) in 51 articles. A description of the coding of the moderators is provided below.

#### Sexual Health Domains and Subdomains

To examine whether educational differences vary according to the domain of sexual health, a categorical variable was created to indicate whether the sexual health outcomes in the studies reflect (1) sexual behavior, (2) sexual attitudes, or (3) sexual knowledge. Within these broad domains, more specific subdomains were also coded. Regarding sexual behavior, the following subdomains were included: early first intercourse (coded for studies with samples with a mean age younger than 16), has had sex (coded for studies with samples with a mean age of or older than 16), has had multiple sex partners, contraceptive use, condom use, has had STI test, has had STI, has experienced pregnancy, pornography use, online sexual risk behavior / sexting, and has experienced sexual violence. Additionally, sexual behavior measures that were reported in fewer than three independent samples (Van Eldik et al., 2020) were grouped into a residual category; Other subdomains of sexual behavior (e.g., problems with sexual functioning, having had an abortion, and sexual violence perpetration). Regarding sexual attitudes, the following subdomains were included: positive attitudes, and the residual category Other subdomains of sexual attitudes (e.g., attitudes about homosexuality, permissive sexual attitudes, and STI risk perception). Regarding sexual knowledge, the following subdomains were included: STI knowledge and the residual category Other subdomains of sexual knowledge (e.g., knowledge about sexual health in general, and about emergency contraception).

#### Stage of Adolescence

To examine whether the stage of adolescence moderates the overall effect, each study was categorized and coded as (1) early adolescence (10–13 years), (2) middle adolescence (14–16 years) and late adolescence (17–19 years). This categorization was based on the sample’s age range, and, alternatively, in studies that either spanned multiple stages of adolescence or did not provide an age range, the sample’s mean age. Age was also examined as a continuous moderator, centering the mean age of participants of each study around its pooled mean (i.e., 16.59 years); however, model fit was comparable, and stage of adolescence was retained due to its stronger grounding in developmental theory and greater interpretability.

#### Gender

To assess the moderating role of participants’ gender on the overall effect, the proportions of male, female and non-binary participants (range from 0 to 1) of each study were coded as continuous variables. However, due to the limited number of studies that reported data on non-binary participants, it was only possible to include the proportion of male and female participants in the moderation analysis.

#### Socioeconomic Status

To examine whether participants’ socioeconomic status (SES) moderates the overall effect, each study was categorized and coded as (1) ≥ 75% of participants with a low SES, (2) ≥ 75% of participants with a middle or high SES, or (3) mixed SES sample (neither group ≥ 75%). This categorization was based on different indicators provided by the studies, namely: parental education, income or occupation, or self-rated family financial situation.

#### Ethnic Background

To examine whether participants’ ethnic background moderates the overall effect, each study was coded as (1) ≥ 75% of participants with a migration background, (2) ≥ 75% without a migration background, or (3) no dominant category (neither group ≥ 75%). Participants were categorized as having a migration background when they themselves and/or their parents were born in another country than the one where the study took place.

#### Geographical Scope

To assess whether the geographical scope of the study moderates the overall effect, the studies were categorized and coded as (1) participants were sampled in one region (e.g., province, state), or (2) participants were sampled in multiple regions.

#### (Timing of) Educational Tracking

As the included studies were conducted in countries with differing educational systems with varying (numbers of) educational tracks, cross-national comparability was enabled by categorizing and coding the educational tracks of each country as either vocational or academic based on the International Standard Classification of Education (ISCED; UNESCO Institute for Statistics, 2012). When studies reported results separately for three or more educational tracks, sample-size-weighted calculations were used to obtain a single estimate per vocational and academic track. Additionally, to examine whether the timing of educational tracking moderates the overall effect, the tracking of the educational system in the studied country was categorized as (1) early (before 14 years) or (2) late (at or after 14 years).

#### Publication Year

To test whether the year in which a study was published moderates the overall effect, the publication year of each study was coded as a continuous variable and centered around its mean (i.e., 2015). A value of 0 indicated the mean year of publication, while positive and negative values reflected the number of years above or below the mean, respectively (e.g., a study published in 2017 was coded as + 2, and one from 2012 as -3).

#### Study Quality

To examine whether the quality of the included studies moderates the overall effect, studies were assessed using the Standard Quality Assessment Criteria for Evaluating Primary Research Papers from a Variety of Fields (QualSyst) (Kmet et al., 2004). QualSyst originally contains 14 items, but for the current study 11 were included due to the (non-interventional) design of the included studies. Each item was scored to indicate whether the study fulfilled the criteria (0 = no, 1 = partial, 2 = yes). Aspects such as the study design, subject selection, sample size and analytic methods were evaluated. Details can be found in the supplementary materials. The scores for the 11 items were summed to obtain a total score, which was converted to a percentage score (i.e., total study score divided by total maximum possible score of 22) and rated “excellent” (> 80%), “good” (70%–79%), “adequate” (55%–69%), “low” (< 55%) (Fang et al., 2020).

### Effect Size Calculations

To examine the relationship between educational track and sexual health among adolescents in Europe, Hedges’ *g*, which is a small-sample correction of Cohen’s *d* (Hedges & Olkin, 1985), was employed as the effect size in the meta-analysis. Individual effect size estimates were computed for each outcome. Different statistics were used to calculate Hedges’ *g* and the corresponding standard error and variance. The majority of studies provided percentages or means, standard deviations, and sample sizes, or other effect sizes, such as correlations and odds ratios. Only unadjusted effect sizes were included (e.g., educational effects examined in multiple regression analyses were not included). The computations were performed with the online practical meta-analysis effect size calculator (Wilson, 2023). All outcomes were (re)coded so that higher positive scores meant that attending vocational tracks, compared to attending academic tracks, is associated with suboptimal sexual health outcomes. The effect sizes were weighted by the inverse variance to ensure that samples with more precise estimates got a greater weight in the analyses (Marín-Martínez & Sánchez-Meca, 2010). An effect size of *g* = 0.2 is considered as small, of *g* = 0.5 as medium, and of *g* = 0.8 as large (Tomczak & Tomczak, 2014).

### Statistical Analyses

Because several articles reported multiple effect sizes, a three-level meta-analysis was conducted to account for possible dependencies among effect sizes. In a three-level meta-analysis, effect sizes are treated as nested within studies, allowing simultaneous estimation of sampling variance, within-study variance, and between-study variance, thereby accounting for the statistical dependence among multiple effect sizes reported within the same study (Van den Noortgate et al., 2013). This study conducted a three-level meta-analysis using the Metafor package (Version 4.8-0) in R (Assink & Wibbelink, 2016; Viechtbauer, 2010). In the main effects analysis, restricted maximum likelihood (REML) estimation was used for estimating the parameters in the model. To identify potential outliers, *z*-scores were computed for all effect sizes. Effect sizes with *z*-scores that were more than three standard deviations from the mean were considered a potential outlier. Overall heterogeneity was assessed with the *Q*- and *I*^*2*^-methods. To evaluate the levels at which heterogeneity appeared, significance of within- and between-study variances was tested by conducting one-sided log-likelihood-ratio tests. The proportion of variance attributed to each level was computed using formulas described by Cheung (2014; as cited in Assink & Wibbelink, 2016). Further moderation analyses were conducted to analyze the sources of heterogeneity. Moderators were examined when at least three independent samples provided information (Van Eldik et al., 2020). Cases with missing data on a particular moderator variable were excluded from the analysis of that moderator. Both categorical (e.g., sexual health domain, SES) and continuous (e.g., publication year, age) variables were tested as moderators. For categorical moderators, dummy variables were created for the categories and added to the overall model. To examine publication bias, three approaches were employed: funnel plots, Egger’s regression method (Egger et al., 1997) and the trim-and-fill method (Duval & Tweedie, 2000). In the context of the funnel plot, an asymmetrical distribution may suggest the presence of publication bias (Borenstein et al., 2009). Regarding Egger’s regression method, an insignificant result signifies an absence of publication bias (Egger et al., 1997). The trim-and-fill method estimates the number of missing studies due to publication bias and adjusts the meta-analysis accordingly (Duval & Tweedie, 2000). Sensitivity analysis was conducted to assess the robustness of the meta-analysis results, using the Leave-One-Out method, where each study was removed sequentially to examine their influence on the overall result (Harrer et al., 2021).

## Results

### Characteristics of Included Studies

This meta-analysis included 47 studies, which provided 159 effect sizes. Details concerning the characteristics of the included studies are summarized in Tables 1 and 2. Studies from 21 different European countries were included, with most studies conducted in Sweden and the Netherlands (both 11), followed by Germany (6), and Italy (4). The majority of studies (32 studies; 68.1%) collected data in multiple regions (e.g., provinces, states) and 15 studies (31.9%) only in one region. In 19 studies (40.4%), participants were divided into educational tracks before they were 14 years old, in 28 studies (59.6%) this occurred at or after 14 years. There were 45 (95.7%) cross-sectional studies and two (4.3%) longitudinal studies. Studies included a total of 177,169 participants from independent samples, ranging from *n* = 192 to 48,504 participants per study. All studies had nonclinical, community-based samples. The mean pooled age of participants is 16.59 years (*SD* = 2.17). The majority of studies (18 studies; 38,3%) included participants in the stages of both middle and late adolescence (14–19 years), followed by 10 studies (21,3%) focusing on late adolescence only (17–19 years), 9 studies (19,1%) including participants of all stages of adolescence (10–19 years), 5 studies (10,6%) focusing on middle adolescence (14–16 years) and the same amount (10,6%) including participants in the stages of both early and middle adolescence (10–16 years), and no studies focusing on early adolescence only (10–13 years). Five studies (10.6%) included only female participants, and two studies (4.3%) included only male participants. Of the 23 studies that reported participants’ ethnic background, 16 studies (69.6%) had samples with over 75% participants without a migration background, 7 studies (30.4%) had samples with a mix of participants with and without a migration background (with no group exceeding 75%), and no studies had samples with over 75% participants with a migration background. Of the 12 studies that reported participants’ SES, 3 studies (25%) had samples with over 75% middle or high SES participants, 9 studies (75%) had samples with a mix of middle or high and low SES participants (with no group exceeding 75%), and no studies had samples with over 75% low SES participants. Sexual behavior variables (e.g., having had sex, condom use) were examined most often, being included in 37 studies (78.7%), followed by sexual attitude variables (e.g., evaluation of first sexual intercourse, STI risk perception) in 12 studies (25.5%) and sexual knowledge variables (e.g., knowledge about STIs) in 10 studies (21.3%). All studies used self-report measures, and two (4.3%) also included records measures (specifically Chlamydia Trachomatis infection tests).

**Table 1:** Characteristics of the Included Studies

Authors (year)	Sample size	Country	Mean age	Age range	Gender (% male)	SES	Ethnic background	Geographical representation	Timing educational tracking
Aho et al. (2016)	5960	Sweden	17.3	16–20	50.4	> 75% middle / high	> 75% majority	Multiple regions	Late
Algren et al. (2022)	28,620	Denmark	-	15–25	47.2	**-**	**-**	Multiple regions	Late
Babjakova et al. (2019)	525	Slovakia	17.18	15–19	38	**-**	> 75% majority	1 region	Late
Baumgartner et al. (2012)	1762	Netherlands	14.52	12–18	51	Mixed	**-**	Multiple regions	Early
Beckmann et al. (2021)	3974	Germany	15	-	46.6	> 75% middle / high	Mixed	1 region	Early
Boahene et al. (2022)	566	Netherlands	17.17	16–20	42	**-**	**-**	Multiple regions	Early
Brunelli et al. (2022)	747	Italy	14.8	14–16	58.8	> 75% middle / high	> 75% majority	1 region	Late
Cerbara et al. (2023)	4288	Italy	16	-	58.8	**-**	> 75% majority	Multiple regions	Late
Czerwinski et al. (2018)	4714	Poland	18.7	-	42.3	**-**	**-**	Multiple regions	Late
De Graaf et al. (2009)	2616	Netherlands	-	12–24	48.3	**-**	> 75% majority	Multiple regions	Early
De Graaf et al. (2015)	7705	Netherlands	-	12–24	49.9	**-**	> 75% majority	Multiple regions	Early
De Looze et al. (2012)	5422	Netherlands	13.83	12–16	50.8	**-**	Mixed	Multiple regions	Early
De Looze et al. (2013)	1568	Netherlands	14.6	13–17	44	**-**	-	Multiple regions	Early
Deleré et al. (2013)	2001	Germany	-	18–20	0	**-**	-	Multiple regions	Early
Delva et al. (2008)	597	Bosnia and Herzegovina, FYR of Macedonia, Serbia, Montenegro	16.7	12–24	-	**-**	-	Multiple regions	Late
Easton et al. (2004)	2615	Hungary	-	15–18	52.2	**-**	-	1 region	Late
Elmerstig et al. (2013)	1566	Sweden	18.3	18–22	0	**-**	> 75% majority	Multiple regions	Late
Falah-Hassani et al. (2007)	6590	Finland	-	16–18	0	Mixed	-	Multiple regions	Late
Fredlund et al. (2017); Jonsson et al. (2019)	5750	Sweden	18	-	44	> 75% middle / high	Mixed	Multiple regions	Late
Gravningen et al. (2012; 2013)	1031	Norway	17.2	15–20	45.2	-	Mixed	1 region	Late
Häggström-Nordin et al. (2011)	384	Sweden	16.1	15-20	46	-	-	Multiple regions	Late
Holmberg & Hellberg (2007)	1332	Sweden	17.3	16-18	57.9	Mixed	> 75% majority	1 region	Late
Kastbom et al. (2014); Jonsson et al. (2014)	2649	Sweden	18.3	-	46.4	-	> 75% majority	Multiple regions	Late
Kuortti & Kosunen (2009)	247	Finland	16.75	15–18	0	-	-	1 region	Late
Larsson et al. (2007); Häggström-Nordin et al. (2009)	718	Sweden	18	17–21	53.9	-	-	1 region	Late
Makenzius et al. (2009)	192	Sweden	18	-	100	-	> 75% majority	1 region	Late
Matić Bojić & Bovan (2024)	1122	Croatia	-	17–19	52.2	-	-	Multiple regions	Late
Mattebo et al. (2013)	477	Sweden	16.5	-	100	-	-	Multiple regions	Late
Patseadou et al. (2010)	523	Greece	16.82	15–18	43.7	Mixed	> 75% majority	1 region	Late
Priebe & Svedin (2008)	1493	Sweden	18.17	-	-	Mixed	Mixed	Multiple regions	Late
Rachamin et al. (2023)	1076	Switzerland	13.8	13–15	47.3	-	-	1 region	Late
Romito & Beltramini (2015)	702	Italy	18.2	-	46	-	-	Multiple regions	Late
Rummel et al. (2022)	3834	Germany	15.26	12–17	47.9	-	-	Multiple regions	Early
Samkange-Zeeb et al. (2013a; 2013b)	1148	Germany	15	12–20	45	Mixed	Mixed	1 region	Early
Schouten et al. (2007)	481	Netherlands	15.9	-	42.4	**-**	> 75% majority	Multiple regions	Early
Seiler-Ramadas et al. (2020)	198	Austria	-	13–16	54.5	Mixed	Mixed	1 region	Early
Sekera et al. (2020)	1942	Czech Republic	16.4	15–19	55.3	**-**	**-**	Multiple regions	Late
Stiekema et al. (2024)^a^	742	Netherlands	13.6	12–16	44.3	**-**	> 75% majority	Multiple regions	Early
Stiekema et al. (2024)^b^	892	Netherlands	13.7	12–16	45.3	**-**	> 75% majority	Multiple regions	Early
Van Lisdonk et al. (2015)	305	Netherlands	16.75	16–18	39	**-**	> 75% majority	Multiple regions	Early
Van Rossem et al. (2010)	11,872	Belgium	15.5	-	48.5	Mixed	> 75% majority	Multiple regions	Early
Vanden Abeele et al. (2014)	1943	Belgium	15.28	11–20	50.6	**-**	**-**	Multiple regions	Early
Veldwijk et al. (2012)	48,504	Netherlands	14.4	13–16	49	**-**	**-**	Multiple regions	Early
Visalli et al. (2014)	1588	Italy	-	16–18	42.9	**-**	**-**	1 region	Late
Von Rosen et al. (2018)	1177	Germany	14.6	13–16	52.52	**-**	Mixed	1 region	Early
Witkowska & Gådin (2005)	540	Sweden	-	17–18	0	**-**	**-**	Multiple regions	Late
Young et al. (2017)	1751	England	-	16–19	46	**-**	> 75% majority	Multiple regions	Late

**Table 2:** Characteristics of the Included Studies

Authors (year)	k	Sexual health domain(s)	Sexual health subdomain(s)	Reporter	Study design
Aho et al. (2016)	1	Behavior	Has experienced sexual violence	S	C
Algren et al. (2022)	1	Behavior	Condom use	S	C
Babjakova et al. (2019)	1	Behavior	Has had sex (sample > 16)	S	C
Baumgartner et al. (2012)	2	Behavior	Online sexual risk behavior / sexting, Other subdomains sexual behavior (Casual sex)	S	L
Beckmann et al. (2021)	1	Behavior	Other subdomains sexual behavior (Sexual violence perpetration)	S	C
Boahene et al. (2022)	1	Behavior	Has had sex (sample > 16)	S	C
Brunelli et al. (2022)	1	Knowledge	Other subdomains sexual knowledge (Sexual health knowledge)	S	C
Cerbara et al. (2023)	1	Behavior	Pornography use	S	C
Czerwinski et al. (2018)	7	Behavior Knowledge	Has had sex (sample > 16), Has had multiple sex partners, Condom use Other subdomains sexual knowledge (STI information sources)	S, R	C
De Graaf et al. (2009)	1	Behavior	Other subdomains sexual behavior (Progressive sexual trajectory)	S	C
De Graaf et al. (2015)	10	Behavior Attitudes	Early first intercourse (sample < 16), Has had multiple sex partners, Has experienced sexual violence, Condom use, Contraception use, Has experienced pregnancy, Has had STI, Other subdomains sexual behavior (Problems with sexual functioning) Evaluation of first intercourse	S	C
De Looze et al. (2012)	1	Behavior	Early first intercourse (sample < 16)	S	C
De Looze et al. (2013)	2	Behavior Attitudes	Early first intercourse (sample < 16) Other subdomains sexual attitudes (Perception of sex as adult behavior)	S	C
Deleré et al. (2013)	3	Behavior Knowledge	Other subdomains sexual behavior (HPV vaccination) STI knowledge	S	C
Delva et al. (2008)	1	Behavior	Has had STI test	S	C
Easton et al. (2004)	10	Behavior	Has experienced sexual violence, Has had sex (sample > 16), Has had multiple sex partners, Early first intercourse (sample < 16), Condom use, Contraceptive use, Has experienced pregnancy, Other subdomains sexual behavior (STI teaching in school)	S	C
Elmerstig et al. (2013)	1	Behavior	Other subdomains sexual behavior (To continue vaginal intercourse despite pain)	S	C
Falah-Hassani et al. (2007)	1	Knowledge	Other subdomains sexual knowledge (Awareness of emergency contraceptive)	S	L
Fredlund et al. (2017); Jonsson et al. (2019)	2	Behavior	Has experienced sexual violence, Other subdomains sexual behavior (Using sex as self-injury)	S	C
Gravningen et al. (2012; 2013)	3	Behavior	Has had STI, Has had STI test	S, R	C
Häggström-Nordin et al. (2011)	4	Behavior	Has had sex (sample > 16), Pornography use, Contraceptive use, Has had STI	S	C
Holmberg & Hellberg (2007)	7	Behavior	Has had sex (sample > 16), Contraceptive use, Has had multiple sex partners, Has experienced pregnancy, Has had STI	S	C
Kastbom et al. (2014); Jonsson et al. (2014)	2	Behavior	Early first intercourse (sample < 16), Online sexual risk behavior / sexting	S	C
Kuortti & Kosunen (2009)	1	Behavior	Has had multiple sex partners	S	C
Larsson et al. (2007); Häggström-Nordin et al. (2009)	11	Behavior Attitudes	Has had sex (sample > 16), Contraceptive use, Has had STI, Other subdomains sexual behavior (Has had abortion, Alcohol use and intercourse) Positive attitudes (Sexual satisfaction, Evaluation of first and latest intercourse)	S	C
Makenzius et al. (2009)	13	Behavior Attitudes Knowledge	Has had sex (sample > 16), Early first intercourse (sample < 16), Has had multiple sex partners, Condom use, Contraceptive use, Has had STI test, Other subdomains sexual behavior (Alcohol use and intercourse) Other subdomains sexual attitudes (Attitude about sex on first date, Attitude about sex with friends, Attitude about girls taking initiative) STI knowledge	S	C
Matić Bojić & Bovan (2024)	1	Attitudes	Other subdomains sexual attitudes (Attitude about homosexuality)	S	C
Mattebo et al. (2013)	1	Behavior	Pornography use	S	C
Patseadou et al. (2010)	1	Behavior	Has had sex (sample > 16)	S	C
Priebe & Svedin (2008)	1	Behavior	Other subdomains sexual behavior (Disclosure of sexual abuse)	S	C
Rachamin et al. (2023)	4	Behavior Attitudes	Has experienced sexual violence Other subdomains sexual attitudes (Feeling informed about sexuality)	S	C
Romito & Beltramini (2015)	1	Behavior	Pornography use	S	C
Rummel et al. (2022)	1	Attitudes	Other subdomains sexual attitudes (Interest in HPV vaccination)	S	C
Samkange-Zeeb et al. (2013a; 2013b)	8	Knowledge Attitudes	STI knowledge, Other subdomains sexual attitudes (STI risk perception)	S	C
Schouten et al. (2007)	6	Behavior Attitudes	Other subdomains sexual behavior (Parent-adolescent communication about sex) Other subdomains sexual attitudes (Beliefs about parent-adolescent communication about sex)	S	C
Seiler-Ramadas et al. (2020)	13	Behavior Attitudes	Other subdomains sexual behavior (Information sources used) Other subdomains sexual attitudes (Feeling informed about sexuality)	S	C
Sekera et al. (2020)	1	Knowledge	STI knowledge	S	C
Stiekema et al. (2024)^a^	3	Attitudes	Other subdomains sexual attitudes (Attitude about homosexuality)	S	C
Stiekema et al. (2024)^b^	3	Attitudes	Other subdomains sexual attitudes (Attitude about homosexuality)	S	C
Van Lisdonk et al. (2015)	1	Behavior	Has had sex (sample > 16)	S	C
Van Rossem et al. (2010)	2	Behavior Knowledge	Early first intercourse (sample < 16) STI Knowledge	S	C
Vanden Abeele et al. (2014)	2	Behavior	Online sexual risk behavior / sexting, Pornography use	S	C
Veldwijk et al. (2012)	1	Behavior	Has experienced sexual violence	S	C
Visalli et al. (2014)	1	Knowledge	STI knowledge	S	C
Von Rosen et al. (2018)	17	Knowledge	STI knowledge	S	C
Witkowska & Gådin (2005)	1	Behavior	Has experienced sexual violence	S	C
Young et al. (2017)	1	Behavior	Has experienced sexual violence	S	C

Abbreviations: *k* = Number of effect sizes, S = Self-report; R = Record; C=Cross - Sectional; L = Longitudinal

### Quality Assessment

The final overall scores ranged from 45.5% to 100%, with a total of 31 studies rated as “excellent”, 8 studies rated as “good”, 5 studies rated as “adequate”, and 3 studies rated as “low”. Detailed quality assessment scores are provided in the supplementary materials. Study quality was included as a moderator.

### Overall Association and Heterogeneity Analysis

**Fig. 2:**
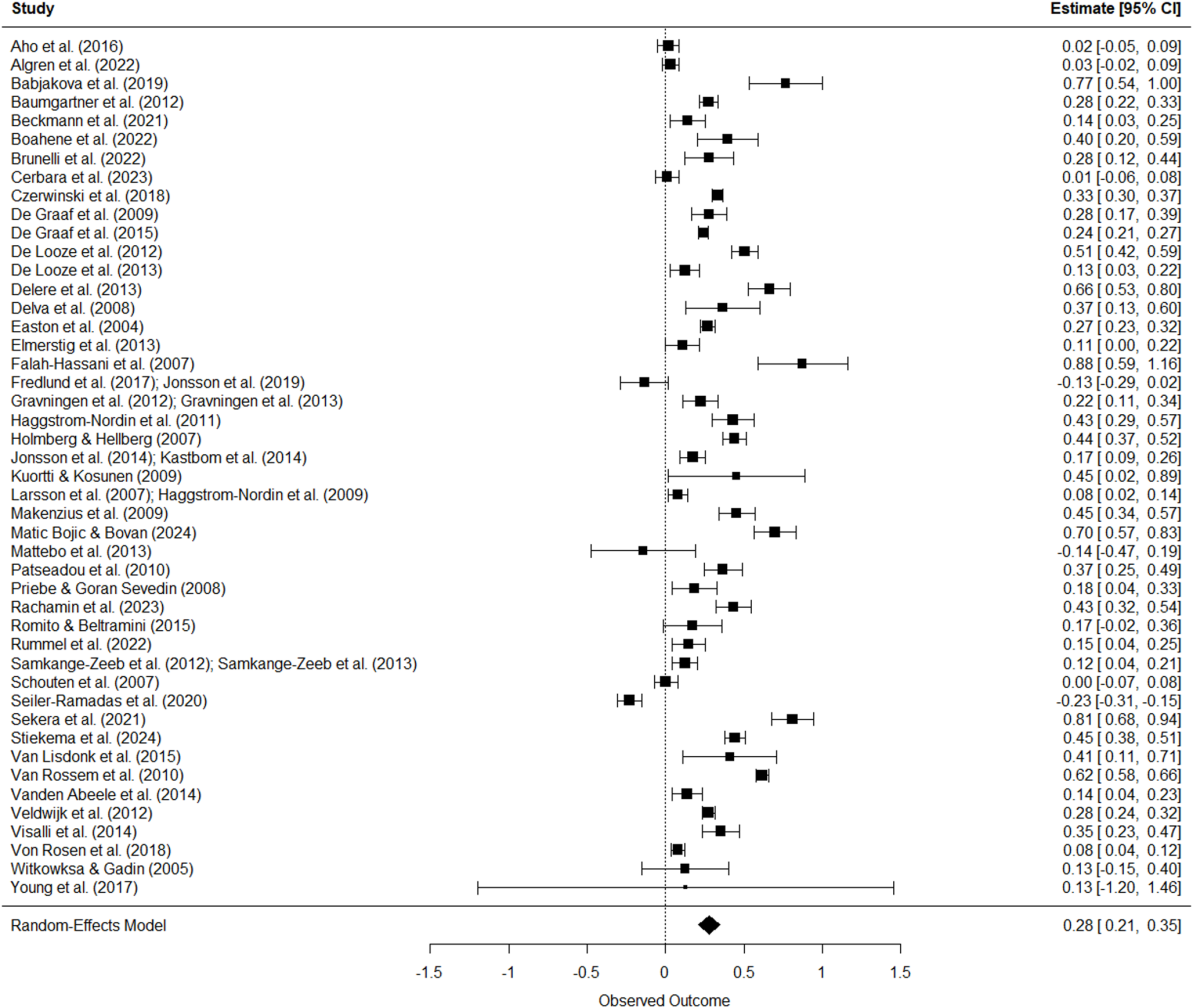
Forest Plot of Effect Sizes of the Association of Educational Track and Sexual Health Included in the Meta-Analysis

The main effect analysis, conducted with the three-level random-effects model described above, revealed a significant positive association between educational track and adolescent sexual health (Hedges’ *g* = 0.279; 95% CI[0.204, 0.354]; *p* < 0.001) (see Fig. 2), indicating that attending vocational tracks, compared to academic tracks, is associated with suboptimal outcomes in the included sexual health indicators overall. According to the criteria formulated by Tomczak and Tomczak (2014), the overall effect of 0.279 can be regarded as small to medium. No effect sizes deviated more than three standard deviations from the mean effect size, indicating that no outliers were detected.

Testing revealed significant heterogeneity across the effect sizes (*Q*(158) = 1960.297, *p* < 0.001). Additionally, an *I*^*2*^ value of 80.23%, falling below the 75% threshold for variance attributable to sampling variance (Hunter & Schmidt, 1990), indicated substantial variation in effect sizes within studies and/or between studies. It was examined how the total variance in observed effect sizes was decomposed into sampling variance, within-study and between-study variance. There was significant within-study variance (σ² = 0.053, 95% CI[0.039, 0.074], *p* < 0.001), which accounted for 58.44% of the total variance. There was also significant between-study variance (σ² = 0.033, 95% CI[0.016, 0.066], *p* < 0.001), which accounted for 36.12% of the total variance. Additionally, sampling variance accounted for 5.43% of the total variance. As most of the variance lies within and between studies rather than in sampling error, this implies that the included studies differ substantially in design, measurements, population characteristics, and/or contextual factors. Thus, the pooled estimate should be interpreted with caution as an average of highly diverse effects, rather than a single, precise estimate of the true effect. In summary, both the heterogeneity test and the variance contribution percentages indicated significant heterogeneity among the included studies, warranting further moderation analyses to explain the sources of variation.

### Moderation

Table 3 presents the results of the moderating variables. Significant moderation effects were found for the subdomains of sexual behavior and sexual attitudes and for the ethnic background of the sample. The moderating effects of the domain of sexual health, the subdomain of sexual knowledge, and of other sample and study characteristics (the gender, stage of adolescence, and SES of the participants, the geographical scope, the timing of educational tracking, publication year, study quality) were not significant.

**Table 3:** Moderation Analysis Results

Moderator variable	No. studies	No. ES	g (SE)	t value (sig)	95% CI	F(df1, df2)
*Sexual health domains*						
Sexual behavior	37	93	0.247^a^(0.042)	5.894***	[0.164, 0.329]	F (2,156) = 2.802
Sexual attitudes	12	35	0.264^ab^ (0.064)	4.140***	[0.138, 0.390]	*p* = 0.064
Sexual knowledge	10	31	0.436^b^ (0.076)	5.711***	[0.285, 0.587]	
*Sexual behavior subdomains*						
Has experienced pregnancy	3	3	0.495^a^ (0.110)	4.508***	[0.276, 0.713]	F(11, 81) = 4.941***
Has had sex (sample *≥* 16)	11	13	0.470^a^ (0.058)	8.126***	[0.355, 0.586]	p>0.001
Has had STI test	3	4	0.447^ab^ (0.125)	3.576***	[0.198, 0.695]	
Early first intercourse (sample < 16)	6	6	0.384^abc^ (0.088)	4.386***	[0.210, 0.558]	
Has had multiple sex partners		7	0.316^abc^ (0.076)	4.147***	[0.164, 0.468]	
Has had STI	6	6	0.230^abc^ (0.096)	2.389*	[0.038, 0.422]	
Contraceptive use	6	8	0.177^bc^ (0.072)	2.469*	[0.034, 0.319]	
Pornography use	5	5	0.150^bc^ (0.096)	1.558	[-0.042, 0.341]	
Online sexual risk behavior / sexting	3	3	0.136^bc^ (0.103)	1.319	[-0.069, 0.341]	
Other subdomains sexual behavior	13	24	0.119^c^ (0.051)	2.311*	[0.016, 0.221]	
Has experienced sexual violence	8	9	0.102^c^ (0.071)	1.446	[-0.038, 0.243]	
Condom use	5	5	0.083^c^ (0.081)	1.025	[-0.078, 0.244]	
*Sexual attitudes subdomains*						
Positive attitudes	3	5	-0.087 (0.164)	-0.535	[-0.420, 0.245]	F(1, 33) = 4.463*
Other subdomains sexual attitudes	11	30	0.284 (0.086)	3.285**	[0.108, 0.459]	*p* = 0.042
*Sexual knowledge subdomains*						
STI Knowledge	6	27	0.515 (0.129)	3.996***	[0.251, 0.779]	F(1,29) = 0.002
Other subdomains sexual knowledge	3	4	0.526 (0.203)	2.591*	[0.111, 0.942]	*p* = 0.964
*Gender (percentage of male participants)*	46	158	0.378 (0.104)	3.646***	[0.173, 0.584]	F(1, 156) = 1.080 *p* = 0.300
*Stage of adolescence*						
Early adolescence (10–13)	4	11	0.464 (0.128)	3.618***	[0.211, 0.717]	F (2, 156) = 2.374
Middle adolescence (14–16)	21	77	0.203 (0.053)	3.833***	[0.098, 0.308]	*p* = 0.096
Late adolescence (17–19)	22	71	0.321 (0.053)	6.012***	[0.215, 0.426]	
*Ethnic background*						
Mix with/without migration background	7	43	0.058 (0.083)	0.703	[-0.106, 0.233]	F (1, 96) = 7.461**
Without migration background	17	55	0.339 (0.061)	5.578***	[0.219, 0.460]	*p* = 0.008
*Socio-economic status*						
Mix low and middle/high SES	8	37	0.282 (0.116)	2.430*	[0.047, 0.517]	F(1, 39) = 0.027
Middle/high SES	3	4	0.239 (0.232)	1.032	[-0.230, 0.708]	*p* = 0.870
*Timing of educational tracking*						
Before 14 years	20	79	0.247 (0.056)	4.409***	[0.137, 0.358]	F(1, 157) = 0.589
At or after 14 years	27	80	0.306 (0.051)	5.954***	[0.204, 0.407]	*p* = 0.444
*Geographical scope*						
One region	15	92	0.255 (0.061)	4.208***	[0.136, 0.375]	F(1, 157) = 0.255
Multiple regions	32	67	0.295 (0.049)	6.031***	[0.198, 0.391]	*p* = 0.614
*Publication year*	47	159	-0.002 (0.007)	-0.276	[-0.015, 0.012]	F(1, 157) = 0.076 *p* = 0.783
*Study quality*						
Excellent	32	94	0.270 (0.048)	5.665***	[0.176, 0.364]	F(3, 155) = 0.309
Good	7	30	0.289 (0.096)	3.017**	[0.100, 0.478]	*p* = 0.819
Adequate	5	32	0.258 (0.108)	2.400*	[0.046, 0.471]	
Low	3	3	0.445 (0.181)	2.463*	[0.088, 0.802]	

Note. The regression coefficients for the categorical variables can be interpreted as the mean effect sizes for each category. ES = effect sizes; *g* = Hedges’ *g*, regression coefficient; CI = confidence interval. Effect sizes are significantly different (*p* < 0.05) if they do not have the same subscript

*p* < 0.05, **p < 0.01; ***p < 0.001

Although the domain of sexual health (sexual behavior, sexual attitudes, or sexual knowledge) did not significantly moderate the association between educational track and adolescent sexual health (*F*(2, 156) = 2.802, *p* = 0.064), inspection of the effect sizes suggested that the domain of sexual knowledge showed a comparatively larger effect. Post hoc exploratory pairwise comparisons confirmed a significant difference between sexual behavior (*g* = 0.247, 95% CI [0.164, 0.329]) and sexual knowledge (*g* = 0.436, 95% CI [0.285, 0.587]). This suggests that the differences between students in vocational and academic tracks are potentially larger when it comes to their sexual knowledge than their sexual behavior, with adolescents attending vocational tracks especially having less knowledge about sexual health than those attending academic tracks. No significant differences were found between sexual attitudes and the other sexual health domains.

Within the different domains of sexual health, analyses were conducted to estimate effect sizes for more specific subdomains. A significant moderation effect was found within the sexual behavior domain (*F*(11, 81) = 4.941, *p* < 0.001). Among the 12 subdomains of sexual behavior that could be analyzed, the following eight showed significant small to medium mean effect sizes, suggesting that students in vocational tracks, compared to those in academic tracks, more often reported: “has had sex”, “early first intercourse”, “has had multiple sex partners”, “has had STI”, “has experienced pregnancy”, and “other subdomains of sexual behavior”, and less often reported “contraceptive use” and “has had STI test”. Secondly, a significant moderation effect was also found within the sexual attitudes domain (*F*(1, 33) = 4.463, *p* = 0.042). A non-significant mean effect size was found for “positive attitudes” (*g* = -0.087, 95% CI [-0.420, 0.245]), and a small to medium significant mean effect size for “other subdomains of sexual attitudes” (*g* = 0.284, 95% CI [0.108, 0.459]), suggesting that adolescents in vocational tracks, compared to those in academic tracks, had for example somewhat more negative attitudes about homosexuality, more permissive sexual attitudes, and lower STI risk perception. No significant moderation effect was found within the sexual knowledge domain.

Lastly, the moderating effect of ethnic background of the sample was significant (*F*(1, 96) = 7.461, *p* = 0.008). A non-significant mean effect size was found for studies with a sample with a mix of participants with and without a migration background (*g* = 0.058, 95% CI [-0.106, 0.233]), and a small to medium significant mean effect size for studies with a sample of more than 75% participants without a migration background (*g* = 0.339, 95% CI [0.219, 0.460]). This suggests that the association between educational track and adolescent sexual health was significantly stronger in studies in which more than 75% of participants did not have a migration background, whereas this difference was not found in studies with a sample with a mix of participants with and without a migration background.

However, heterogeneity remained significant after the individual moderator variables were added to the meta-analytic model, as well as after testing a multiple moderator model including the moderators that were significant in the univariate analyses (see Table 4), indicating that a single moderator as well as the multiple moderator model could not explain all variance.

**Table 4:** Heterogeneity Test of Moderator Variables

Moderator variable	Q	df	*p*	I^2^
Sexual health domains	1875.036	156	< 0.001	79.70
Sexual behavior subdomains	506.828	81	< 0.001	65.34
Sexual attitudes subdomains	290.205	33	< 0.001	82.56
Sexual knowledge subdomains	461.122	29	< 0.001	87.42
Gender	1950.567	156	< 0.001	80.47
Stage of adolescence	1882.920	156	< 0.001	79.33
Ethnic background	1182.886	96	< 0.001	76.28
Socio-economic status	707.984	39	< 0.001	89.01
Timing of educational tracking	1956.177	157	< 0.001	80.24
Geographical scope	1893.464	157	< 0.001	80.34
Publication year	1906.623	157	< 0.001	80.46
Study quality	1755.976	155	< 0.001	80.70
Multiple moderator model of significant moderators (Sexual behavior subdomains, Sexual attitudes subdomains, Ethnic background)	748.176	84	< 0.001	72.59

### Publication Bias and Sensitivity Analysis

**Fig. 3:**
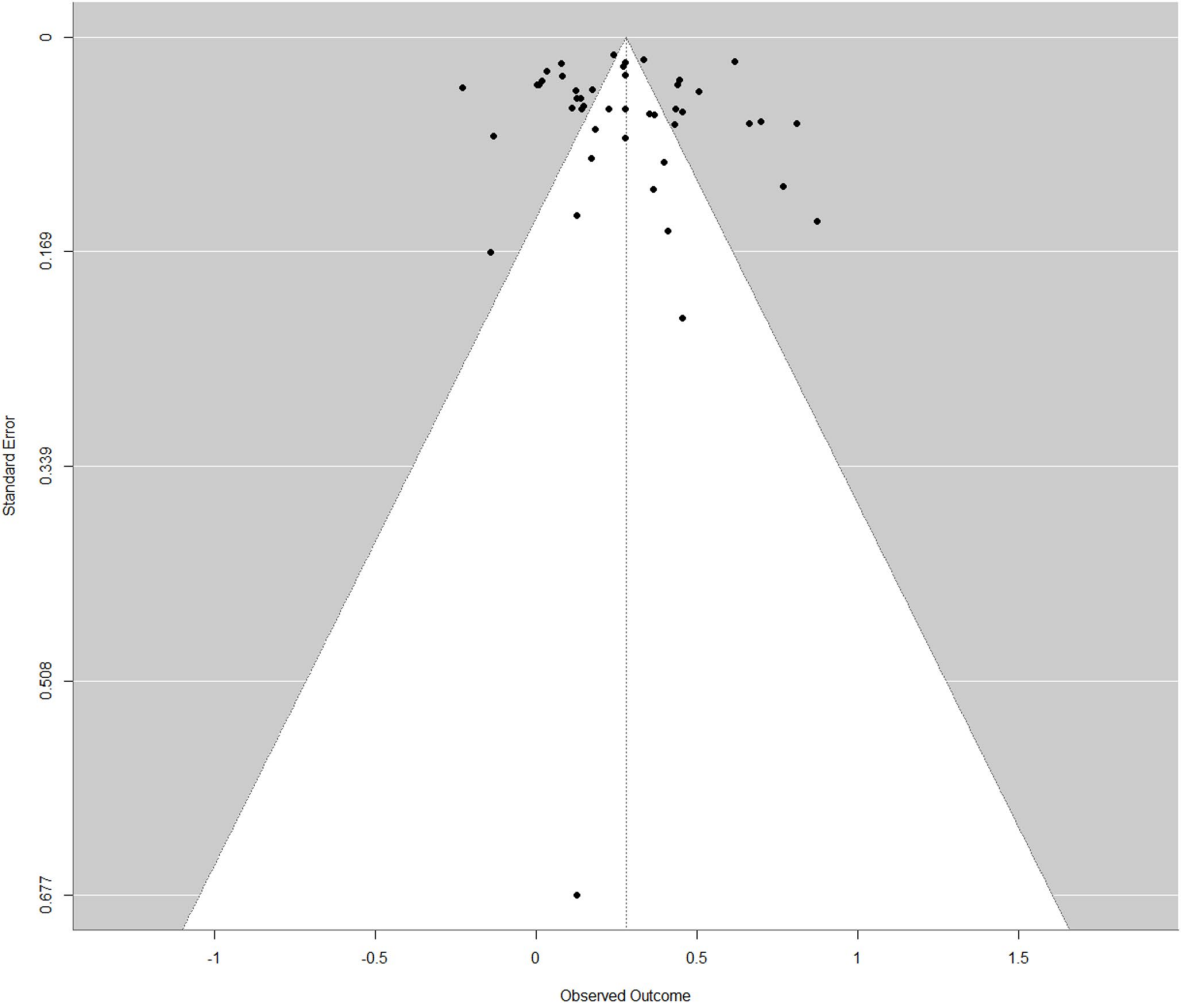
Funnel plot for Association between Educational Track and Sexual Health

Publication bias refers to the phenomenon in which significant results are more likely to be published, leading to a distortion of meta-analytic findings from the true effect (Rosenthal, 1979). Publication bias was first assessed visually using a funnel plot (Fig. 3), which appeared nearly symmetrical. Egger’s test results suggested that publication bias may have been present (*t* = 2.1873, *p* = 0.0302), but trim-and-fill analysis estimated that no (zero) studies may be missing due to publication bias. Thus, no strong evidence of publication bias was found.

Leave-one-out sensitivity analyses excluded each study and reconducted the meta-analysis. The effect size estimates varied minimally, ranging from 0.2668 to 0.2914, without affecting the significance of the result. These findings indicated that the meta-analysis was not substantially influenced by any single study, confirming the robustness and reliability of the conclusions.

## Discussion

Although various studies have explored the relationship between educational track and adolescent sexual health, important gaps remained due to their focus on single national contexts, assessment of only a limited range of sexual health outcomes, and incomplete examination of key moderating factors, making it difficult to determine the strength of this association and the conditions under which it varies. This study addressed these gaps by synthesizing the available evidence regarding the relationship between educational track and sexual health among adolescents in Europe and exploring potential moderating factors. This meta-analysis summarized 47 studies involving 177,169 adolescents and 159 effect sizes. The results of this meta-analysis showed a significant, small to medium, positive association between educational track and adolescent sexual health, indicating that attending vocational tracks, compared to academic tracks, is associated with suboptimal outcomes in the included sexual health indicators overall. More specifically, educational differences varied according to the subdomains of sexual behavior and sexual attitudes, and to the ethnic background of the sample. No significant effects were found for the other moderators, that is, the broader domains of sexual health, the subdomain of sexual knowledge, and the gender, stage of adolescence, and SES of the participants, the geographical scope, the timing of educational tracking, publication year, and study quality.

### Heterogeneity

One key finding of the meta-analysis is the high heterogeneity across the included studies, which is not uncommon in meta-analytic research, particularly when combining studies from different national contexts with varying educational systems, study designs, and populations. While heterogeneity itself is not inherently problematic, it does require careful consideration, especially when interpreting the overall effect size. The variance decomposition analysis indicates that a substantial portion of the variability lies within and between studies, rather than being attributable to sampling error alone. This suggests that the studies differ significantly in terms of design, measurement, and/or contextual factors, such as differences in the operationalization of educational tracking, sexual health measures, and the demographics of the study populations.

Despite testing various moderators - including study quality, sample characteristics, and contextual factors - these moderators explain only a small portion of the observed heterogeneity. Substantial variability remained unexplained across studies, suggesting other unmeasured factors, such as cultural differences and local (sexuality) educational practices, likely influence the relationship between educational track and adolescent sexual health.

Given the high heterogeneity, readers should interpret the overall pooled effect size with caution. A majority of the included studies did find a positive association between educational track and adolescent sexual health, indicating that the variance is mostly regarding the strength of the effect, rather than the direction. While the results provide valuable insights into the general trends and associations between educational track and adolescent sexual health, the significant heterogeneity indicates that these relationships may not be universal or consistent across all contexts. The findings should be viewed as indicative rather than definitive, highlighting broad patterns while acknowledging the limitations of generalizability across diverse educational and cultural contexts. In future research, it would be beneficial to explore the sources of this heterogeneity in greater depth, particularly by examining country-specific factors or educational policies that could affect the relationship between educational track and sexual health outcomes. Until then, the current study provides an important step in understanding the overarching trends, but it also underscores the need for caution when extrapolating these findings to all educational systems or populations.

### Educational Differences in (Sub)Domains of Sexual Health

When comparing the three broader domains of sexual health, that is, sexual behavior, sexual attitudes, and sexual knowledge, the overall moderating effect was not significant. However, follow-up analyses indicated that, compared to sexual behavior, educational differences were significantly larger for sexual knowledge, with adolescents attending vocational tracks having less knowledge about sexual health than those attending academic tracks. When comparing the subdomains of sexual knowledge, the moderating effect was not significant, indicating that the educational differences were similar across different types of sexual knowledge. As has been suggested in previous research, it is possible that differences in sex education play a role in the educational differences in sexual knowledge. It has been argued that adolescents in vocational tracks might not receive the same quality and quantity of sex education as young people in academic tracks, both at school (De Graaf et al., 2015) and at home (Schouten et al., 2007), or that mainstream sex education programs are less tailored to the needs of students in vocational tracks (Van Rossem et al., 2010).

When zooming in on the domain of sexual attitudes, only a comparison between positive attitudes and a residual category was possible, as too few studies reported on other specific subdomains. The largest educational differences were found for this residual category. Students in vocational tracks had for example somewhat more negative attitudes about homosexuality, more permissive sexual attitudes, and lower STI risk perception compared to students in academic tracks, but no educational differences were found for positive attitudes.

When comparing the different subdomains of sexual behavior, the largest educational differences were found for having had sex (at an early age) and having experienced pregnancy, with students in vocational tracks more often having had sex (at an early age) and having experienced pregnancy than students in academic tracks. Other significant educational differences were found for having been tested for STIs and having had an STI, having had multiple sex partners, contraceptive use, and the residual category for sexual behavior, which for example included sexual violence perpetration and problems with sexual functioning. In contrast, no significant educational differences were found for condom use, having experienced sexual violence, pornography use, and online risk behavior or sexting.

### Educational Differences in Sexual Health Across Different Contexts

The association between educational track and adolescent sexual health varied significantly depending on the ethnic background of the sample, with stronger effects found for samples with mostly participants without a migration background. This may be because for participants with a migration background, other factors than educational track might show stronger associations with sexual health, such as culture or religion (Cense, 2014; Sinha et al., 2007). Alternatively, it could be that participants with a migration background face discrimination and structural barriers in accessing sexual health resources (Wayal et al., 2017), which could produce differences even within the same educational track. Aside from this finding about ethnic background, no other study and sample characteristics significantly moderated the association between educational track and adolescent sexual health, implying that this relationship remains similar across varying study and sample characteristics.

The non-significant effect of gender contrasts with some previous research findings showing the relationship between educational track and adolescent sexual health to be stronger for girls (Czerwinski et al., 2018; De Graaf et al., 2015). As this study explored the moderating effect using gender ratios, and with most studies maintaining the gender ratio at around 50%, this might have hindered the revelation of gender differences in the relationship between educational track and adolescent sexual health. Therefore, the gender differences in this relationship still deserve verification in future research.

### Study Contributions

This study makes several important contributions to the understanding of the relationship between educational track and sexual health among adolescents in Europe. First, by conducting a three-level meta-analysis, possible dependencies among effect sizes could be accounted for, thereby increasing the robustness of the findings (Van den Noortgate et al., 2013). Second, this study confirmed that there are educational differences in sexual health among adolescents in Europe. Specifically, students in vocational tracks have less knowledge about sexual health and tend to initiate sexual activity at an earlier age than students in academic tracks, thereby becoming exposed to certain risks, such as STIs and unintended pregnancies, at a younger age. These results highlight the need for earlier and tailored sex education for students in vocational tracks. The finding that most of the moderator variables were non-significant further emphasizes the critical role of school-level interventions in reducing sexual health disparities. Importantly, though, by examining sexual health as a multidimensional concept consistent with the definition of the World Health Organization (2010), this study demonstrates that there are also aspects of sexual health (such as positive attitudes, condom use, experiencing sexual violence and online sexual risk behavior or sexting) that show no significant educational differences. Thus, tailored sex education programs for students in vocational tracks can, on the one hand, address specific issues and thereby help reduce inequalities. On the other hand, caution should be exercised not to focus exclusively on differences, as students in vocational and academic tracks also share many similarities when it comes to their sexual health. Lastly, these results also highlight the need for future research on adolescent sexual health to move beyond a solely risk-oriented focus and to adopt a broader perspective to achieve a more comprehensive understanding of sexual health disparities.

### Limitations and Future Recommendations

The findings of this study should be interpreted in the light of its limitations. Although a thorough and systematic search was conducted across a variety of databases, there may be other databases that possibly could have resulted in additional findings. Second, given that only published studies were included and the search was restricted to English- and Dutch-language articles, it is possible that relevant literature is missing from the current study. Finally, a number of articles could not be included in the meta-analysis, as they did not provide sufficient statistical information to calculate effect sizes. The authors of those articles were not contacted; this could have helped to include more studies in the meta-analysis.

Reviewing the literature on educational differences in adolescent sexual health yielded several suggestions for future research. Firstly, it is recommended for future studies to examine broader, and especially more positive or emotional aspects of sexual health, as these appeared to be largely missing from the literature. Second, future research should focus on identifying the factors that contribute to educational differences in sexual health. For example, it is important to investigate the extent to which sex education and sexual health services adequately address the needs of students in vocational tracks. Further, over half of the studies included in the present meta-analysis did not report participants’ SES (35 studies; 74.5%) or ethnic background (24 studies; 51.1%). Of those studies that did, none included samples composed primarily of participants of low SES or with a migration background. Additionally, young adolescents were underrepresented in the present meta-analysis, with no studies focusing solely on early adolescence (10–13 years). Researchers are strongly encouraged to report these demographic characteristics in future studies, and to prioritize the inclusion of underrepresented populations. Furthermore, greater attention to gender and sexual diversity in future research is warranted, as currently only a limited number of studies have examined these factors, preventing the possibility of inclusion in the moderation analyses of the present study. Finally, it is also a possibility that a bidirectional relationship exists between educational track and adolescent sexual health, with for example studies showing teen pregnancy and educational attainment each influencing the other (Högnäs & Grotta, 2019; Olausson et al., 2001). Future research should employ longitudinal designs to explore the causal relationship between educational track and sexual health aspects other than teen pregnancy.

## Conclusion

Several studies have explored educational differences among adolescents in various aspects of sexual health, but uncertainty remained about how large the differences in sexual health between adolescents in different educational tracks are, and whether these differences vary according to study and sample characteristics such as gender, ethnic background or SES. This study addresses this gap by conducting a three-level meta-analysis, which revealed a significant positive association between educational track and adolescent sexual health. Specifically, it was found that educational differences were largest regarding sexual knowledge. Additionally, students in vocational tracks were more likely to have had sex (at an early age) and have experienced pregnancy, but there were no differences in a number of other aspects of sexual health, such as positive attitudes, condom use and experiencing sexual violence. With respect to study and sample characteristics, it was found that educational differences are larger for studies with mostly participants without a migration background. No other study and sample characteristics significantly moderated the association between educational track and adolescent sexual health. These results imply that school-level interventions, with earlier and tailored sex education for students in vocational tracks, represent an important opportunity for reducing sexual health disparities. At the same time, an exclusive focus on differences should be avoided, as students in vocational and academic tracks also share many similarities when it comes to their sexual health. Equipping adolescents, both in vocational and academic tracks, with the knowledge and skills necessary to prevent adverse outcomes and enhance their sexual health is essential.

## Supplementary Information

Below is the link to the electronic supplementary material.

Supplementary Material 1

Supplementary Material 2

Supplementary Material 3

## Data Availability

Data, materials and code are available at the DANS Data Station Social Sciences and Humanities repository: https://doi.org/10.17026/SS/VTM0SO.
