# Supplementary material for: Educational Differences in Sexual Health among Adolescents in Europe: A Meta-Analysis
Source: J Youth Adolesc. 2026 Jun 8;55(8):1943–63. doi: 10.1007/s10964-026-02364-3 (PMC13442477; doi:10.1007/s10964-026-02364-3)
Supplement: Supplementary file 1 [file 10964_2026_2364_MOESM1_ESM.docx]

Educational Differences in Sexual Health among Adolescents in Europe:

A Meta-Analysis

Journal of Youth and Adolescence

**Online Resource 2 – Classification Educational Tracks per Country**

| **Country** | **Timing of tracking  (age)** | **Educational track** | **Classification** | **Studies** |
| --- | --- | --- | --- | --- |
| Austria | 10 | Academic secondary school | Academic | Seiler-Ramadas et al. (2020) |
|  |  | Vocational secondary school | Vocational |  |
| Belgium | 13 | ASO (general secondary education) | Academic | Van Rossem et al. (2010)  Vanden Abeele et al. (2014) |
|  |  | KSO (arts secondary education) | Academic |  |
|  |  | TSO (technical secondary education) | Academic |  |
|  |  | BSO (vocational secondary education) | Vocational |  |
| Bosnia and Herzegovina, FYR of Macedonia, Serbia, Montenegro | 15 | General secondary school  Vocational secondary school | Academic  Vocational | Delva et al. (2008) |
| Croatia | 14 | Grammar school | Academic | Matić Bojić & Bovan (2024) |
|  |  | Art school | Academic |  |
|  |  | Vocational school | Vocational |  |
| Czech Republic | 15 | General secondary education | Academic | Sekera et al. (2021) |
|  |  | Vocational secondary education | Vocational |  |
| Denmark | 16 | General secondary education | Academic | Algren et al. (2022) |
|  |  | Vocational secondary education | Vocational |  |
| England | 16 | Academic education | Academic | Young et al. (2017) |
|  |  | Vocational education | Vocational |  |
| Finland | 16 | General secondary education | Academic | Falah-Hassani et al. (2007)  Kuortti & Kosunen (2009) |
|  |  | Vocational secondary education | Vocational |  |
| Germany | 10 | Gymnasium (grammar school)  Gesamtschule (comprehensive school)  Realschule (intermediate school)  Hauptschule (general secondary school) | Academic  Academic  Academic  Vocational | Beckmann et al. (2021)  Deleré et al. (2013)  Rummel et al. (2022)  Samkange-Zeeb et al. (2013a)  Samkange-Zeeb et al. (2013b)  Von Rosen et al. (2018) |
|  |  |  |  |  |
|  |  |  |  |  |
|  |  |  |  |  |
| Greece | 15 | General secondary school | Academic | Patseadou et al. (2010) |
|  |  | Vocational secondary school | Vocational |  |
| Hungary | 14 | General secondary school | Academic | Easton et al. (2004) |
|  |  | Vocational secondary school | Vocational |  |

| **Country** | **Timing of tracking  (age)** | **Educational track** | **Classification** | **Studies** |
| --- | --- | --- | --- | --- |
| Italy | 14 | General secondary education  Technical secondary education  Vocational secondary education | Academic  Academic  Vocational | Brunelli et al. (2022)  Cerbara et al. (2023)  Romito & Beltramini (2015)  Visalli et al. (2014) |
|  |  |  |  |  |
|  |  |  |  |  |
| The Netherlands | 12 | VWO (pre-university secondary education)  HAVO (general secondary education)  VMBO (pre-university secondary education) | Academic  Academic  Vocational | Baumgartner et al. (2012)  Boahene et al. (2022)  De Graaf et al. (2009)  De Graaf et al. (2015)  De Looze et al. (2012)  De Looze et al. (2013)  Schouten et al. (2007)  Stiekema et al. (2024)  Van Lisdonk et al. (2015)  Veldwijk et al. (2012) |
| Norway | 16 | General secondary education | Academic | Gravningen et al. (2012)  Gravningen et al. (2013) |
|  |  | Vocational secondary education | Vocational |  |
| Poland | 15 | General secondary education | Academic | Czerwinski et al. (2018) |
|  |  | Vocational secondary education | Vocational |  |
| Slovakia | 15 | Gymnasium (general secondary education) | Academic | Babjakova et al. (2019) |
|  |  | Conservatory (arts secondary education) | Academic |  |
|  |  | Vocational secondary education | Vocational |  |
| Sweden | 16 | General secondary education  Vocational secondary education | Academic  Vocational | Aho et al. (2016)  Elmerstig et al. (2013)  Fredlund et al. (2017)  Häggström-Nordin et al. (2011)  Holmberg & Hellberg (2007)  Jonsson et al. (2014)  Larsson et al. (2007)  Makenzius et al. (2009)  Mattebo et al. (2013)  Priebe & Göran Svedin (2008)  Witkowska & Gådin (2005) |
|  |  |  |  |  |
| Switzerland | 15 | Baccalaurate (academic secondary education) | Academic | Rachamin et al. (2023) |
|  |  | Specialised secondary school | Academic |  |
|  |  | Vocational secondary education | Vocational |  |
