# Supplementary material for: Educational Differences in Sexual Health among Adolescents in Europe: A Meta-Analysis
Source: J Youth Adolesc. 2026 Jun 8;55(8):1943–63. doi: 10.1007/s10964-026-02364-3 (PMC13442477; doi:10.1007/s10964-026-02364-3)
Supplement: Supplementary file 2 [file 10964_2026_2364_MOESM2_ESM.docx]

Educational Differences in Sexual Health among Adolescents in Europe:

A Meta-Analysis

Journal of Youth and Adolescence

**Online Resource 3 – Study Quality Assessment**

| **Study** | **Criteria** | | | | | | | | | | | **Total scores** | **Category** |
| --- | --- | --- | --- | --- | --- | --- | --- | --- | --- | --- | --- | --- | --- |
|  | **Q1** | **Q2** | **Q3** | **Q4** | **Q8** | **Q9** | **Q10** | **Q11** | **Q12** | **Q13** | **Q14** |  |  |
| Aho et al. (2016) | 2 | 2 | 2 | 2 | 2 | 2 | 2 | 2 | 2 | 2 | 2 | 22 | Excellent |
| Algren et al. (2022) | 2 | 2 | 2 | 2 | 1 | 2 | 2 | 2 | 2 | 2 | 2 | 21 | Excellent |
| Babjakova et al. (2019) | 1 | 2 | 1 | 2 | 0 | 1 | 1 | 2 | 0 | 2 | 1 | 13 | Adequate |
| Baumgartner et al. (2012) | 2 | 2 | 2 | 2 | 2 | 2 | 2 | 2 | 1 | 2 | 2 | 21 | Excellent |
| Beckmann et al. (2021) | 2 | 2 | 2 | 2 | 2 | 2 | 2 | 2 | 2 | 2 | 2 | 22 | Excellent |
| Boahene et al. (2022) | 2 | 2 | 1 | 2 | 2 | 2 | 1 | 2 | 2 | 2 | 2 | 20 | Excellent |
| Brunelli et al. (2022) | 1 | 2 | 1 | 2 | 1 | 2 | 1 | 2 | 2 | 2 | 1 | 17 | Good |
| Cerbara et al. (2023) | 2 | 2 | 2 | 2 | 2 | 2 | 2 | 2 | 2 | 2 | 2 | 22 | Excellent |
| Czerwinski et al. (2018) | 2 | 2 | 1 | 1 | 1 | 1 | 2 | 2 | 2 | 2 | 2 | 18 | Excellent |
| De Graaf et al. (2009) | 2 | 1 | 1 | 2 | 1 | 2 | 2 | 2 | 2 | 2 | 2 | 19 | Excellent |
| De Graaf et al. (2015) | 2 | 2 | 2 | 2 | 2 | 2 | 2 | 1 | 2 | 1 | 2 | 20 | Excellent |
| De Looze et al. (2012) | 2 | 2 | 2 | 2 | 2 | 2 | 2 | 2 | 2 | 2 | 2 | 22 | Excellent |
| De Looze et al. (2013) | 2 | 2 | 1 | 1 | 2 | 2 | 2 | 2 | 2 | 2 | 2 | 20 | Excellent |
| Deleré et al. (2013) | 2 | 2 | 1 | 1 | 1 | 2 | 1 | 2 | 2 | 2 | 2 | 18 | Excellent |
| Delva et al. (2008) | 1 | 2 | 2 | 1 | 1 | 2 | 2 | 2 | 2 | 1 | 2 | 18 | Excellent |
| Easton et al. (2004) | 2 | 2 | 2 | 1 | 1 | 2 | 1 | 2 | 1 | 1 | 2 | 17 | Good |
| Elmerstig et al. (2013) | 2 | 2 | 1 | 2 | 2 | 2 | 2 | 2 | 2 | 2 | 2 | 21 | Excellent |
| Falah-Hassani et al. (2007) | 2 | 2 | 2 | 2 | 2 | 2 | 2 | 2 | 2 | 2 | 2 | 22 | Excellent |
| Fredlund et al. (2017) | 2 | 2 | 1 | 2 | 2 | 2 | 2 | 2 | 2 | 2 | 2 | 21 | Excellent |
| Gravningen et al. (2012) | 2 | 2 | 1 | 2 | 1 | 0 | 2 | 2 | 2 | 2 | 1 | 17 | Good |
| Gravningen et al. (2013) | 2 | 2 | 1 | 2 | 1 | 2 | 2 | 2 | 2 | 2 | 2 | 20 | Excellent |
| Kastbom et al. (2014) | 2 | 2 | 2 | 2 | 2 | 2 | 2 | 2 | 2 | 1 | 1 | 20 | Excellent |
| Kuortti & Kosunen (2009) | 2 | 2 | 1 | 1 | 1 | 1 | 2 | 2 | 2 | 1 | 2 | 17 | Good |
| Larsson et al. (2007) | 1 | 2 | 2 | 1 | 0 | 1 | 2 | 2 | 2 | 2 | 2 | 17 | Good |
| Makenzius et al. (2009) | 2 | 2 | 2 | 0 | 1 | 1 | 1 | 2 | 0 | 1 | 2 | 14 | Adequate |
| Matić Bojić & Bovan (2024) | 2 | 2 | 2 | 1 | 2 | 2 | 2 | 2 | 2 | 2 | 2 | 21 | Excellent |
| Mattebo et al. (2013) | 2 | 2 | 1 | 1 | 1 | 1 | 2 | 2 | 2 | 1 | 1 | 17 | Good |
| Patseadou et al. (2010) | 2 | 2 | 2 | 2 | 1 | 2 | 2 | 2 | 2 | 1 | 2 | 20 | Excellent |
| Priebe & Svedin (2008) | 1 | 2 | 1 | 2 | 2 | 2 | 1 | 2 | 2 | 2 | 2 | 19 | Excellent |
|  |  |  |  |  |  |  |  |  |  |  |  |  |  |

| **Study** | **Criteria** | | | | | | | | | | | **Total scores** | **Category** |
| --- | --- | --- | --- | --- | --- | --- | --- | --- | --- | --- | --- | --- | --- |
|  | **Q1** | **Q2** | **Q3** | **Q4** | **Q8** | **Q9** | **Q10** | **Q11** | **Q12** | **Q13** | **Q14** |  |  |
| Rachamin et al. (2023) | 2 | 2 | 2 | 1 | 1 | 2 | 2 | 1 | 1 | 1 | 1 | 16 | Good |
| Romito & Beltramini (2015) | 2 | 2 | 2 | 2 | 2 | 2 | 1 | 2 | 2 | 1 | 2 | 20 | Excellent |
| Rummel et al. (2022) | 2 | 2 | 1 | 1 | 1 | 2 | 1 | 0 | 0 | 1 | 1 | 12 | Adequate |
| Samkange-Zeeb et al. (2013a) | 2 | 2 | 1 | 2 | 2 | 2 | 2 | 2 | 2 | 2 | 1 | 20 | Excellent |
| Samkange-Zeeb et al. (2013b) | 2 | 2 | 2 | 2 | 1 | 2 | 1 | 2 | 1 | 1 | 2 | 18 | Excellent |
| Schouten et al. (2007) | 2 | 2 | 0 | 2 | 1 | 1 | 2 | 2 | 2 | 2 | 2 | 18 | Excellent |
| Seiler-Ramadas et al. (2020) | 1 | 2 | 1 | 2 | 1 | 1 | 1 | 2 | 2 | 1 | 1 | 15 | Adequate |
| Sekera et al. (2021) | 1 | 2 | 1 | 1 | 1 | 2 | 1 | 0 | 0 | 1 | 1 | 11 | Low |
| Stiekema et al. (2024) | 2 | 2 | 1 | 2 | 2 | 2 | 2 | 2 | 2 | 2 | 2 | 21 | Excellent |
| Van Lisdonk et al. (2015) | 2 | 2 | 1 | 2 | 1 | 2 | 2 | 0 | 2 | 2 | 2 | 18 | Excellent |
| Van Rossem et al. (2010) | 1 | 2 | 2 | 1 | 1 | 2 | 1 | 2 | 2 | 1 | 2 | 17 | Good |
| Vanden Abeele et al. (2014) | 2 | 2 | 2 | 1 | 2 | 2 | 1 | 2 | 2 | 2 | 2 | 20 | Excellent |
| Veldwijk et al. (2012) | 2 | 2 | 1 | 2 | 1 | 2 | 2 | 2 | 2 | 1 | 2 | 19 | Excellent |
| Visalli et al. (2014) | 1 | 2 | 1 | 0 | 1 | 2 | 1 | 0 | 0 | 1 | 1 | 10 | Low |
| Von Rosen et al. (2018) | 2 | 2 | 1 | 2 | 1 | 2 | 2 | 2 | 2 | 1 | 2 | 19 | Excellent |
| Witkowska & Gådin (2005) | 1 | 1 | 2 | 1 | 1 | 2 | 1 | 0 | 0 | 1 | 1 | 11 | Low |
| Young et al. (2017) | 2 | 2 | 1 | 2 | 2 | 2 | 2 | 2 | 2 | 2 | 1 | 20 | Excellent |

Q1 = Question/objective sufficiently described? ; Q2 = Study design evident and appropriate? ; Q3 = Method of subject/comparison group selection or source of information/input variables described and appropriate? ; Q4 = Subject (and comparison group, if applicable) characteristics sufficiently described? ; Q8 = Outcome and (if applicable) exposure measure(s) well defined and robust to measurement / misclassification bias? Means of assessment reported? ; Q9 = Sample size appropriate? ; Q10 = Analytic methods described/justified and appropriate? ; Q11 = Some estimate of variance is reported for the main results? ; Q12 = Controlled for confounding? ; Q13 = Results reported in sufficient detail? ; Q14 = Conclusions supported by the results?
