# Supplementary material for: Educational Differences in Sexual Health among Adolescents in Europe: A Meta-Analysis
Source: J Youth Adolesc. 2026 Jun 8;55(8):1943–63. doi: 10.1007/s10964-026-02364-3 (PMC13442477; doi:10.1007/s10964-026-02364-3)
Supplement: Supplementary file 3 [file 10964_2026_2364_MOESM3_ESM.docx]

Educational Differences in Sexual Health among Adolescents in Europe:

A Meta-Analysis

Journal of Youth and Adolescence

**Online Resource 1 – Search Terms**

| Concept 1: Educational level | | Concept 2: Sexual health and well-being | | Concept 3: Adolescents | | Concept 4: Countries |
| --- | --- | --- | --- | --- | --- | --- |
| **Subject Headings** | **Free text terms** | **Subject Headings** | **Free text terms** | **Subject Headings** | **Free text terms** | **Free text terms** |
| Pubmed  Educational Status | education level*  education attainment*  education track*  education status  educational level*  educational attainment*  educational track*  educational status  educational achievement*  educational differences  level of education  educated  secondary education  secondary school*  high school*  vocational education  pre-vocational education  prevocational education  academic education  pre-university education | Pubmed  Sexual Health  Sexual Behavior  Sexual Development  Sexual Arousal  Libido  Sexual Dysfunctions, Psychological  Sex Offenses  Erotica  Reproductive Health  Reproductive Behavior  Contraception  Contraceptive Devices  Contraceptive Agents  Sexually Transmitted Diseases  Pregnancy  Abortion, Induced | sexual*  sex behavior  sex behaviour  intercourse  masturbat*  orgasm*  porn*  reproductive  safe sex  unsafe sex  protected sex  unprotected sex  contracepti*  STD  STI  sexually transmitted disease*  sexually transmitted infection*  birth control  condom*  pregnan*  abortion* | Pubmed  Adolescent  Puberty  Minors | adolescen*  youth*  young  teen*  puberty  girl*  boy  boys  minor* | Austria*  Belgi*  Bulgaria*  Croat*  Cyprus  Czech*  Denmark, Danish, Danes  Estonia*  Finland, Finn*  France, French  German*  Greece, Greek  Hungar*  Ireland, Irish  Italy, Italian*  Latvia*  Lithuania*  Luxembourg*  Malta, Maltese  Netherlands, Dutch  Poland, Polish  Portug*  Romania*  Slovak*  Sloven*  Spain, Spanish  Norway, Norwegian*  Switzerland, Swiss  United Kingdom, England, Britain, English, British  Europe* |
| PsycINFO  Educational Attainment Level  Secondary Education  High School Education  High Schools  Vocational Education |  | PsycINFO  Psychosexual Behavior  Psychosexual Development  Sexual Development  Sexual Attitudes  Sexual Intercourse (Human)  Sexual Partners  Sexual Satisfaction  Sex Drive  Sexual Violence  Sexual Reproduction  Reproductive Health  Reproductive Health Care  Sexually Transmitted Diseases  Pregnancy  Adolescent Pregnancy |  | PsycINFO  High School Students  Late Adolescence  Early Adolescence  Puberty |  |  |
